# Effective containment explains subexponential growth in recent confirmed COVID-19 cases in China

**DOI:** 10.1126/science.abb4557

**Published:** 2020-04-08

**Authors:** Benjamin F. Maier, Dirk Brockmann

**Affiliations:** 1 Robert Koch Institute, Nordufer 20, D-13353 Berlin, Germany.; 2 Institute for Theoretical Biology, Humboldt University of Berlin, Philippstrasse 13, D-10115 Berlin, Germany.

## Abstract

The recent outbreak of coronavirus disease 2019 (COVID-19) in mainland China was characterized by a distinctive subexponential increase of confirmed cases during the early phase of the epidemic, contrasting with an initial exponential growth expected for an unconstrained outbreak. We show that this effect can be explained as a direct consequence of containment policies that effectively deplete the susceptible population. To this end, we introduce a parsimonious model that captures both quarantine of symptomatic infected individuals, as well as population-wide isolation practices in response to containment policies or behavioral changes, and show that the model captures the observed growth behavior accurately. The insights provided here may aid the careful implementation of containment strategies for ongoing secondary outbreaks of COVID-19 or similar future outbreaks of other emergent infectious diseases.

## Containment works

National governments have taken different approaches in response to the coronavirus disease 2019 (COVID-19) pandemic, ranging from draconian quarantines to laissez-faire mitigation strategies. In data from China collected in February 2020, Maier and Brockmann noticed that, unexpectedly, the epidemic did not take off exponentially. Nonexponential spread occurs when the supply of susceptible individuals is depleted on a time scale comparable to the infectious period of the virus. The results of the authors' modeling approach indicate that the public response to the epidemic plus containment policies were becoming effective despite the initial increase in confirmed cases.

*Science*, this issue p. 742

## Teaser

Feedback between the epidemic process, the behavioral response to it, and nationwide containment policies can restrain COVID-19.

The outbreak of coronavirus disease 2019 (COVID-19) caused by the coronavirus severe acute respiratory syndrome–coronavirus 2 (SARS-CoV-2) in mainland China was closely monitored by governments, researchers, and the public alike (*1*–*8*). The rapid increase of positively diagnosed cases and subsequent rise of secondary outbreaks in many countries worldwide raised concern on an international scale. The World Health Organization (WHO) therefore announced the COVID-19 outbreak a Public Health Emergency of International Concern on 31 January and eventually classified it as a pandemic on 11 March (*2*, *3*).

In mainland China, confirmed cases increased from ~330 on 21 January 2020 to more than 17,000 on 2 February 2020 within 2 weeks (*9*). In Hubei Province, the epicenter of the COVID-2019 outbreak, confirmed cases rose from 270 to 11,000 in this period; in all other Chinese provinces, the cumulative case count increased from 60 to 6000 in the same period. Yet, as of 28 March, the total case count has saturated at 67,800 cases in Hubei with no new cases per day and reached 13,600 in the remaining Chinese provinces with about 50 new cases per day.

**Fig. 1:**
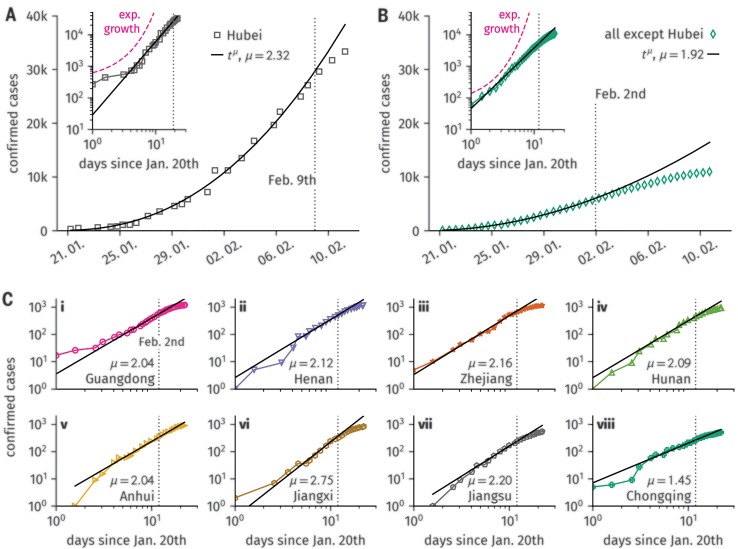
Confirmed cases of COVID-19 infections. *C*(*t*) in mainland China in late January–early February. (**A**) Confirmed case numbers in Hubei. The increase in cases follows a scaling law *t*^µ^ with an exponent µ = 2.32 after a short initial exponential growth phase. On 9 February, the case count starts deviating toward lower values. (**B**) Aggregated confirmed cases in all other affected provinces except Hubei. *C*(*t*) follows a scaling law with exponent µ = 1.92 until 2 February when case counts deviate to lower values. The insets in (A) and (B) depict *C*(*t*) on a log-log scale and show example exponential growth curves for comparison. (**C**) Confirmed cases as a function of time for the eight remaining most affected provinces in China. The curves roughly follow a scaling law with exponents µ ≈ 2 with the exception of Chongqing Province (µ = 1.45) and Jiangxi Province (µ = 2.75).

An initial exponential growth of confirmed cases is generically expected for an uncontrolled outbreak, as observed e.g., during the 2009 influenza A (H1N1) pandemic (*10*) or the 2014 Ebola outbreak in West Africa (*11*). This initial outbreak is in most cases mitigated with a time delay by effective containment strategies and policies that reduce transmission and effective reproduction of the virus, commonly yielding a saturation in the cumulative case count and an exponential decay in the number of new infections (*12*). Although in Hubei the number of laboratory-confirmed cases *C*(*t*) was observed to grow exponentially in early January (*13*), the subsequent rise followed a subexponential, superlinear, algebraic scaling law *t*^µ^ with an exponent μ = 2.3 (between 24 January and 9 February) (compare Fig. 1A). For most of the affected Chinese provinces of mainland China, however, this type of algebraic rise occurred from the beginning of case reporting on 21 January. The exponents μ fluctuate around a typical value of μ = 2.1 ± 0.3 for the confirmed case curves in other substantially affected provinces (confirmed case counts larger than 500 on 12 February), displaying algebraic growth despite geographical, socioeconomical differences, possible differences in containment strategies, and heterogeneities that may have variable impacts on how the local epidemic unfolds (compare Fig. 1, B and C). Eventually, case counts began to deviate from the observed scaling laws around 9 February for Hubei and in early February for the remaining provinces, approaching a saturation behavior.

The appearance of the observed growth behavior for all provinces during the transient phase between onset and saturation suggests that this aspect of the dynamics is determined by fundamental principles that are at work and robust with respect to variation of other parameters that typically shape the temporal evolution of epidemic processes. Three questions immediately arise: (i) What may be the reason for this functional dependency? (ii) Are provinces other than Hubei mostly driven by cases exported from Hubei and therefore follow a similar functional form in case counts, as suggested by preliminary studies discussing the influence of human travel (*14*–*16*)? Or, alternatively, (iii) is the scaling law a consequence of endogenous and basic epidemiological processes, caused by a balance between transmission events and containment efforts?

In the following, we will provide evidence that the implementation of effective containment strategies that target both susceptibles and infecteds can account for the observed growth behavior.

The Chinese government put several mitigation policies in place to suppress the spread of the epidemic (*17*). In particular, positively diagnosed cases were either quarantined in specialized hospital wards or put under a form of monitored self-quarantine at home. Similarly, suspected cases were confined in monitored house arrest, e.g., individuals who arrived from Hubei before all traffic from its capital Wuhan was effectively restricted. These measures aimed at the removal of infectious individuals from the transmission process.

Additionally, introduced social distancing measures aimed at the protection of the susceptible population, induced by behavioral changes as well as the partial shutdown of public life (*17*). For instance, many people wore face masks in public spaces and followed stricter hygiene procedures concerning hand washing; universities remained closed; many businesses closed down; and people were asked to remain in their homes for as much time as possible, in several places enforced by mandatory curfews. Another standard strategy that Chinese authorities applied was contact tracing (*17*), where possible transmission chains between known infecteds and their contacts were identified and suspected cases were isolated at home before symptom onset. Although very effective in interrupting the transmission process and thereby shielding large numbers of susceptibles from acquiring the infection, contact tracing becomes infeasible when the number of infecteds grows rapidly in a short amount of time or when an undetected outbreak leads to a large number of unidentifiable infecteds, as was the case in Hubei.

The latter containment efforts that affect both susceptibles and asymptomatic infectious individuals not only protect susceptibles from acquiring the infection but also remove a substantial fraction of the entire pool of susceptibles from the transmission process, indirectly mitigating the proliferation of the virus in the population in much the same way that herd immunity is effective in the context of vaccine-preventable diseases.

## Modeling epidemic spread under containment efforts

On a very basic level, an outbreak such as the one in Hubei is captured by SIR dynamics where the population is divided into three compartments that differentiate the state of individuals with respect to the contagion process: infected (I), susceptible (S) to infection, and removed (R) (i.e., not taking part in the transmission process) (*18*, *19*). The corresponding variables *S*, *I*, and *R* quantify the respective compartment’s fraction of the total population such that *S* + *I* + *R* = 1. The temporal evolution of the number of cases is governed by two processes: the infection that describes the transmission from an infectious to a susceptible individual with basic reproduction number *R*_0_ and the recovery of an infected after an infectious period of average length *T_I_*. The basic reproduction number *R*_0_ captures the average number of secondary infections an infected will cause before he or she recovers or is effectively removed from the population.

Initially, a small fraction of infecteds yields an exponential growth if the basic reproduction number is larger than unity. A simple reduction in the number of contacts caused by quarantine policies without additional shielding of susceptibles could be associated with a reduction in the effective reproduction number, which would, however, still yield an exponential growth in *I*(*t*) if *R*_0_ > 1, which is inconsistent with the observed transient scaling law *t*^µ^ discussed above. To test the hypothesis that the observed growth behavior can be caused by mitigation policies that apply to both infected and susceptible individuals, we extend the SIR model by two additional mechanisms, one of which can be interpreted as a process of removing susceptibles from the transmission process: First, we assume that general public containment efforts or individual behavioral changes in response to the epidemic effectively remove individuals from the interaction dynamics or substantially reduce their participation in the transmission dynamics. We will refer to this mechanism as “containment” in the following. Second, we account for the removal of symptomatic infected individuals, which we will refer to as “quarantine” procedures. The dynamics are governed by the system of ordinary differential equations

$$\partial_{t}S=-\alpha SI-\kappa_{0}S$$ (1)

$$\partial_{t}I=\alpha SI-\beta I-\kappa_{0}I-\kappa I$$ (2)

$$\partial_{t}R=\beta I+\kappa_{0}S$$ (3)

$$\partial_{t}X=\left(\kappa +\kappa_{0}\right)I$$ (4)

a generalization of the standard SIR model, henceforth referred to as the SIR-X model. The rate parameters α and β quantify the transmission rate and the recovery rate of the standard SIR model, respectively. Additionally, the impact of containment efforts is captured by the terms proportional to the containment rate κ_0_ that is effective in both *I* and *S* populations, because measures such as social distancing and curfews affect the whole population alike. Infected individuals are removed at rate κ corresponding to quarantine measures that only affect symptomatic infecteds. The new compartment *X* quantifies symptomatic, quarantined infecteds. Here we assume that the fraction *X*(*t*) is proportional to the empirically confirmed and reported cases *C*(*t*) and that the time period between sampling and test results can be neglected. The case κ_0_ = 0 corresponds to a scenario in which the general population is unaffected by policies or does not commit behavioral changes in response to an epidemic. The case κ_0_ = 0 corresponds to a scenario in which symptomatic infecteds are not isolated specifically.

In the basic SIR model that captures unconstrained, free spread of the disease, the basic reproduction number *R*_0_ is related to transmission and recovery rate by $R_{0}\equiv R_{0,\mathrm{free}}=\alpha /\beta$ because β^−1^ = *T_I_* is the average time an infected individual remains infectious before recovery or removal. Here, the time period that an infected individual remains infectious is $T_{I,\mathrm{eff}}={\left(\beta +\kappa_{0}+\kappa \right)}^{-1}$ such that the effective, or “observed,” reproduction number $R_{0,\mathrm{eff}}=\alpha T_{I,\mathrm{eff}}$ is smaller than $R_{0,\mathrm{free}}$ because both κ_0_ > 0 and κ > 0.

The key mechanism at work in the model defined by Eqs. 1 to 4 is the exponentially fast depletion of susceptibles in addition to isolation of infecteds. This effect is sufficient to account for the observed scaling law in the number of confirmed cases for a plausible range of model parameters as discussed below.

## Effective protection of susceptibles leads to subexponential growth

**Fig. 2:**
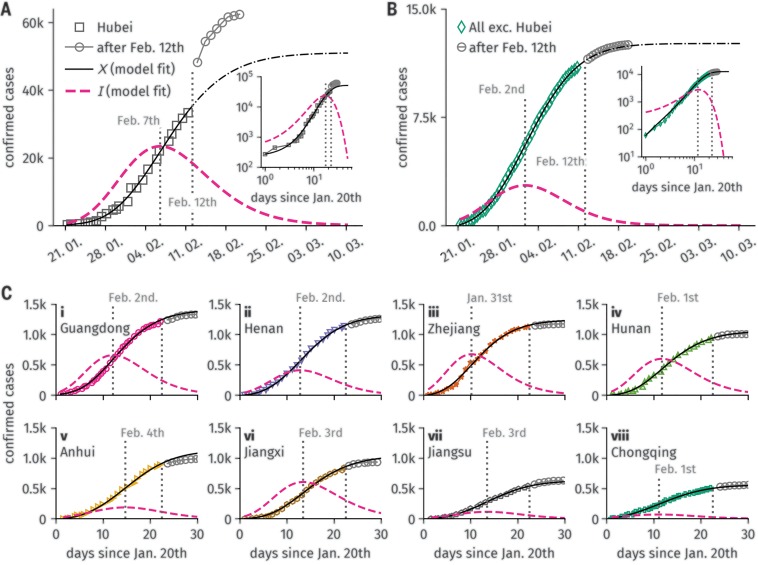
Case numbers in Hubei compared to model predictions. The quarantined compartment *X*(*t*) and the unidentified infectious compartment *I*(*t*) are obtained from fits to the model defined by Eqs. 1 to 4 as described in the materials and methods. All fits were performed for case numbers predating 12 February at which the case number definition was temporarily changed for Hubei, adding ~15,000 cases at once. Consequently, confirmed case numbers after 12 February are well captured by the model for all provinces except Hubei. Fit parameters are given in table S1. (**A**) In Hubei, the model captures both the initial rise of confirmed cases as well as the subsequent algebraic growth. The confirmed cases were predicted to saturate at *C* = 51,000. The model also predicts the time course of the number of unidentified infectious individuals *I*(*t*) which peaks on 7 February and declines exponentially afterwards. Although the magnitude of *I*(*t*) is associated with rather large fluctuations due to uncertainties in the fitting parameters, the predicted peak time is robust, consistently around 7 February. (**B**) Model prediction for case numbers aggregated over all affected provinces other than Hubei. The case numbers’ algebraic growth is well reflected and predicted to saturate at *C* = 12,600. In contrast to Hubei, the fraction of unidentified infecteds peaks around 1 February, ~1 week earlier. The insets in (A) and (B) depict both data and fits on a log-log scale. (**C**) Fits for confirmed cases as a function of time for the remaining eight most affected provinces in China. All curves are well captured by the model fits that predict similar values for the peak time of unidentified infecteds.

We assume that a small number of infected individuals traveled from Hubei to each of the other affected provinces before traffic restrictions were effective but at a time when containment measures were just being implemented. Figure 2 illustrates the degree to which the case count for Hubei Province and the aggregated case count for all other provinces is captured by the SIR-X model as defined by Eqs. 1 to 4.

For a wide range of model parameters, the empirical case count is well reproduced, displaying the observed scaling law *t*^µ^ for a substantial period of time before saturating to a constant level. Notably, the model reproduces both growth behaviors observed in the data: It predicts the expected initial growth of case numbers in Hubei Province followed by an algebraic growth episode for ~11 days until the saturation sets in, a consequence of the decay of unidentified infected individuals after a peak time around 7 February (Fig. 2A). Furthermore, the model also captures the immediate subexponential growth observed in the remaining most affected provinces (Fig. 2, B and C). Again, saturation is induced by a decay of unidentified infecteds after peaks that occur several days before peak time in Hubei, ranging from 31 January to 4 February. For all provinces, following their respective peaks, the number of unidentified infecteds *I*(*t*) decays over a time period that is longer than the reported estimation of maximum incubation period of 14 days (*4*, *20*). It is important to note that the numerical value of unidentified infecteds is sensitive to parameter variations—the general shape of *I*(*t*), however, is robust for a wide choice of parameters, as discussed in the materials and methods.

Parameter choices for best fits were a fixed basic reproduction number of *R*_0,free_ = 6.2 (note that this reproduction number corresponds to an unconstrained epidemic) and a fixed mean infection duration of *T_I _*= 8 days consistent with previous reports concerning the incubation period of COVID-19 (*4*, *20*). The remaining fit parameters are shown in table S1. For these values, the effective basic reproduction number is found to range between 1.4 ≤ *R*_0,eff_ ≤ 3.3 for the discussed provinces, consistent with estimates found in previous early assessment studies (*4*, *8*, *21*, *22*). We discuss these parameters and their possible range in the materials and methods.

On 12 February, case counting procedures were altered regarding the outbreak in Hubei to include cases that were clinically diagnosed and not laboratory confirmed. Consequently, about 15,000 new cases were added on a single day, compared to about 1500 new cases on the day before. We therefore only use data predating 12 February to estimate model parameters for the most affected provinces and compare the obtained predictions with subsequent cases numbers. We find that for all provinces other than Hubei, our predictions accurately reflect the empirical observations (Fig. 2, B and C). When we omit the aforementioned discontinuity that arises in the empirical data, the saturating behavior of cases in Hubei is consistent with the prediction, as well. When the clinically diagnosed cases of 12 February are subtracted from the number of cases in Hubei, our model underestimates the final count of laboratory-confirmed cases in Hubei by 4%. In the remaining part of mainland China, we underestimate the final case count by 7% (as of 28 March).

A detailed analysis of the model parameters indicates that a wide range of values can account for similar shapes of the respective case counts (see materials and methods). Consequently, the model is structurally stable with respect to these parameters, and the numerical value is of less importance than the quality of the mechanism they control. In particular, we find that an exponential decay of available susceptibles is responsible for the observed subexponential growth behavior, i.e., a nonzero containment rate κ_0_ > 0. We present model analyses for all affected Chinese provinces in the supplementary materials (compare figs. S1 and S2, and table S2). We further provide analytical arguments for the emergence of subexponential growth of confirmed cases and derive an approximate expression that relates the scaling-law exponent µ to model parameters, finding reasonable agreement with the empirical values shown in Fig. 1 (compare table S5).

Additionally, we analyzed two model variants in which (i) containment strategies affect the whole population equally (κ_0_ > 0 and κ = 0) and (ii) only infecteds are quarantined (κ = 0 and κ_0_ > 0). The first model captures the case number growth slightly less accurately compared to the complete model (compare figs. S3 and S4, table S3). The second model requires the assumption that shielding susceptibles from transmission was sufficiently effective that only a very small number was at risk of infection, i.e., the effective population size $N_{\mathrm{eff}}\ll N$ becomes an additional model parameter (compare figs. S5 and S6, and table S4). In this case, the immediate exponential depletion of susceptibles because of the transmission process itself causes the empirically observed growth in case numbers. Both of these limiting cases strengthen the point that the fast removal of susceptibles from the population is responsible for the observed subexponential growth.

The described saturation behavior of confirmed cases requires that eventually all susceptibles will effectively be removed from the transmission process. In reality, not every susceptible person can be shielded or shield themselves for such an extended period of time as the model suggests. One might expect instead that the number of unidentified infecteds will decay more slowly and saturate to a small, yet nonzero level, which is why we expect systematic but small underestimations regarding the final empirical case count of epidemic outbreaks.

## Discussion and conclusion

In this study, we find that one of the key features of the dynamics of the COVID-19 epidemic in Hubei Province but also in all other provinces is the robust subexponential rise in the number of confirmed cases according to a scaling law *t*^µ^ during the transient episode of the epidemic before assuming saturating behavior. This general shape of growth suggests that fundamental principles are at work associated with this particular outbreak that are dominated by the interplay of the contagion process with endogenous behavioral changes in the susceptible population and external containment policies. Although the explicit shape of the growth curves discussed here can be influenced by factors such as seasonal effects, systematic delay in reporting, or heterogeneities in demographic structure and population mixing, the total case numbers eventually reached a stable value, which suggests that containment strategies that shielded the susceptible population from the transmission process were rather effective—compared to potential case numbers of an unmitigated outbreak, only a small fraction of the Chinese population that was at risk has been infected to date (29 March). Nevertheless, we cannot rule out that other factors contributed to the growth behavior displayed in the data that were collected over a short period of time during a tense situation.

The model defined by Eqs. 1 to 4 and discussed here indicates that the type of observed growth behavior can generally be expected if the supply of susceptible individuals is systematically decreased by means of implemented containment strategies or behavioral changes in response to information about the ongoing epidemic. Unlike contagion processes that develop without external interference at all or processes that merely lead to parametric changes in the dynamics, our analysis suggests that nonexponential growth is expected when the supply of susceptibles is depleted on a time scale comparable to that of the infectious period of a disease.

The model reproduces the empirical case counts in all provinces well for plausible parameter values. The quality of the reproduction of the case counts in all 29 affected provinces can be used to estimate the peak time of the number of asymptomatic or oligosymptomatic infected individuals in the population, which is the key quantity for estimating the time when an outbreak will wane. The current analysis indicates that this peak time was reached around 7 February for Hubei and within the first days of February in the remaining affected provinces.

The model further suggests that the public response to the epidemic and the containment measures put in place were effective despite the increase in confirmed cases. That this behavior was observed in all provinces also indicates that containment strategies were universally effective. On the basis of our analysis, such strategies would have to stay in effect for a longer time than the maximum incubation period after the saturation in confirmed cases sets in.

Our analysis shows that mitigation strategies that target the susceptible population and induce behavioral changes at this “end” of the transmission process can be very effective in containing an epidemic—especially in situations when asymptomatic or mildly symptomatic infectious periods are long or their duration unknown. Although standard containment strategies such as contact tracing may become infeasible during large-scale outbreaks of such diseases, the implementation of stricter measures can aid in the fast reduction of the number of new infections, thereby quickly increasing the feasibility of interventions that do not affect the general public as drastically. This may be of importance for developing containment strategies for currently developing large-scale secondary outbreaks of COVID-19 in several regions of the world or future outbreaks of other infectious diseases.

We stress that our model describes the general effects of containment mechanisms, effectively averaged over many applied strategies or individual changes of behavior. Our analysis therefore cannot identify the efficacy of specific actions. As the implementation of drastic measures such as mandatory curfews can have severe consequences for both individuals as well as a country’s society and economy, decisions about their application should not be made lightly.

## Supplementary Material

science.sciencemag.org/content/368/6492/742/suppl/DC1 Materials and Methods Supplementary Text Fig. S1 to S6 Tables S1 to S5 Reference (*24*) MDAR Reproducibility Checklist

## Appendix

View/request a protocol for this paper from *Bio-protocol*.

## References

[1] Cohen J., Scientists are racing to model the next moves of a coronavirus that’s still hard to predict. Science (2020); 10.1126/science.abb2161. 10.1126/science.abb2161

[2] WHO, Novel coronavirus (2019-nCoV) situation report - 11 (2020).

[3] WHO, Coronavirus disease 2019 (COVID-19) situation report - 51 (2020).

[4] CDC, 2019 Novel coronavirus (2019-nCoV); https://www.cdc.gov/coronavirus/2019-ncov/about/symptoms.html (accessed 13 February 2020).

[5] Hsu J., Here’s how computer models simulate the future spread of new coronavirus. Sci. Am. (13 February 2020).

[6] Lewis T., China’s citywide quarantines: Are they ethical and effective? Sci. Am. (25 January 2020).

[7] Chen N., Zhou M., Dong X., Qu J., Gong F., Han Y., Qiu Y., Wang J., Liu Y., Wei Y., Xia J., Yu T., Zhang X., Zhang L., Epidemiological and clinical characteristics of 99 cases of 2019 novel coronavirus pneumonia in Wuhan, China: A descriptive study. Lancet 395, 507–513 (2020). 10.1016/S0140-6736(20)30211-7 32007143 PMC7135076

[8] Zhao S., Lin Q., Ran J., Musa S. S., Yang G., Wang W., Lou Y., Gao D., Yang L., He D., Wang M. H., Preliminary estimation of the basic reproduction number of novel coronavirus (2019-nCoV) in China, from 2019 to 2020: A data-driven analysis in the early phase of the outbreak. Int. J. Infect. Dis. 92, 214–217 (2020). 10.1016/j.ijid.2020.01.050 32007643 PMC7110798

[9] Dong E., Du H., Gardner L., An interactive web-based dashboard to track COVID-19 in real time. Lancet (2020). 10.1016/S1473-3099(20)30120-1 PMC7159018 32087114

[10] de Picoli Junior S., Teixeira J. J., Ribeiro H. V., Malacarne L. C., dos Santos R. P., dos Santos Mendes R., Spreading patterns of the influenza A (H1N1) pandemic. PLOS ONE 6, e17823 (2011). 10.1371/journal.pone.0017823 21483857 PMC3069037

[11] Hunt A. G., Exponential growth in Ebola outbreak since May 14, 2014. Complexity 20, 8–11 (2014). 10.1002/cplx.21615

[12] R. M. Anderson, R. M. May, *Infectious Diseases of Humans: Dynamics and Control*, Oxford Science Publications (Oxford Univ. Press, 1991).

[13] Li Q., Guan X., Wu P., Wang X., Zhou L., Tong Y., Ren R., Leung K. S. M., Lau E. H. Y., Wong J. Y., Xing X., Xiang N., Wu Y., Li C., Chen Q., Li D., Liu T., Zhao J., Liu M., Tu W., Chen C., Jin L., Yang R., Wang Q., Zhou S., Wang R., Liu H., Luo Y., Liu Y., Shao G., Li H., Tao Z., Yang Y., Deng Z., Liu B., Ma Z., Zhang Y., Shi G., Lam T. T. Y., Wu J. T., Gao G. F., Cowling B. J., Yang B., Leung G. M., Feng Z., Early Transmission Dynamics in Wuhan, China, of Novel Coronavirus-Infected Pneumonia. N. Engl. J. Med. 382, 1199–1207 (2020). 10.1056/NEJMoa2001316 31995857 PMC7121484

[14] B. Prasse, M. A. Achterberg, L. Ma, P. Van Mieghem, Network-Based Prediction of the 2019-nCoV Epidemic Outbreak in the Chinese Province Hubei; arXiv:2002.04482 [physics, q-bio] (2020). 10.1007/s41109-020-00274-2 PMC7341469 32835088

[15] S. Sanche *et al*, The Novel Coronavirus, 2019-nCoV, is Highly Contagious and More Infectious Than Initially Estimated; arXiv:2002.03268 [q-bio] (2020).

[16] Chen Y., Cheng J., Jiang Y., Liu K., A time delay dynamic system with external source for the local outbreak of 2019-nCoV. Appl. Anal. 1–12 (2020). 10.1080/00036811.2020.1732357

[17] WHO, Report of the WHO-China joint mission on coronavirus disease 2019 (COVID-19) (2020).

[18] M. J. Keeling, P. Rohani, *Modeling Infectious Diseases in Humans and Animals* (Princeton Univ. Press, 2008).

[19] Kermack W. O., McKendrick A. G., Contributions to the mathematical theory of epidemics—I. 1927. Bull. Math. Biol. 53, 33–55 (1991). 2059741 10.1007/BF02464423

[20] WHO, Novel Coronavirus (2019-nCoV) Situation Report - 7 (2020).

[21] A. J. Kucharski *et al.**, *Early dynamics of transmission and control of COVID-19: A mathematical modelling study.* medRxiv* p. 2020.01.31.20019901 (2020); 10.1101/2020.01.31.20019901. PMC7158569 32171059

[22] J. M. Read, J. R. Bridgen, D. A. Cummings, A. Ho, C. P. Jewell, Novel coronavirus 2019-nCoV: Early estimation of epidemiological parameters and epidemic predictions.*****medRxiv* p. 2020.01.23.20018549 (2020); 10.1101/2020.01.23.20018549. 10.1101/2020.01.23.20018549 PMC8165596 34053269

[23] B. F. Maier, benmaier/COVID19CaseNumberModel: Final Analysis; https://zenodo.org/record/3732556 (accessed 29 March 2020). 10.5281/zenodo.3732556

[24] GeoNames Project, GeoNames. https://geonames.org (accessed 1 November 2019).

